# Trajectory shaping guidance for impact angle control of planetary hopping robots

**DOI:** 10.3389/frobt.2024.1452997

**Published:** 2024-11-11

**Authors:** Sabyasachi Mondal, Saurabh Upadhyay

**Affiliations:** Centre for Autonomous and Cyber-Physical Systems, Faculty of Engineering and Applied Sciences, Cranfield University, Cranfield, United Kingdom

**Keywords:** hopping robot, trajectory shaping control, generalized vector explicit (GENEX) guidance, planetary exploration, space robotics

## Abstract

This paper presents a novel optimal trajectory-shaping control concept for a planetary hopping robot. The hopping robot suffers from uncontrolled in-flight and undesired after-landing motions, leading to a position drift at landing. The proposed concept thrives on the Generalized Vector Explicit (GENEX) guidance, which can generate and shape the optimal trajectory and satisfy the end-point constraints like the impact angle of the velocity vector. The proposed concept is used for a thruster-based hopping robot, which achieves a range of impact angles, reduces the position drift at landing due to the undesired in-flight and after-landing motions, and handles the error in initial hopping angles. The proposed approach’s conceptual realization is illustrated by lateral acceleration generated using thruster orientation control. Extensive simulations are carried out on horizontal and sloped surfaces with different initial and impact angle conditions to demonstrate the effect of impact angle on the position drift error and the viability of the proposed approach.

## 1 Introduction

Planetary robots (rovers) play a vital role in planetary surface exploration by gathering terrain information. Wheeled rovers can carry heavy payloads (e.g., scientific instruments, sensors, etc.) and are widely used in planetary exploration. However, they navigate at very low speeds (10 to 20km in 1 year on Mars (Geromichalos et al., 2020)) and cannot traverse rugged terrains. An aerial rover can fly over rugged terrains quickly and provide aerial images. Hence, NASA has tested the first Mars helicopter, Ingenuity, in its Mars 2020 mission (Tzanetos et al., 2022). However, the air density affects aerial vehicles’ operation, and their operation time is short due to high power consumption. Another interesting locomotion proposed for celestial body exploration is hopping (Burdick and Fiorini, 2003). The hopping rovers can traverse rugged terrains while providing aerial and atmospheric data with less power consumption. It has led to significant research in hopping robot design in recent years.

Typically, three design approaches, namely, strike or spring-based, internal flywheel-based, and thruster-based, are proposed to achieve the hopping. The strike or spring-based robots (Burdick and Fiorini, 2003) are the first designs that follow a ballistic trajectory. This robot’s current state-of-the-art uses work manipulation to achieve a jump over 30m height. However, the strike/spring-based designs typically do not have any other mechanism to control the robot’s in-flight and after-landing motions, which degrades the accurate landing performance due to initial launching errors and in-flight uncertainty conditions. The second type of robot considers a cuboid design in general and uses orthogonally placed flywheels for hop (Li et al., 2020; Hockman et al., 2017). These robots can change their attitude in flight, and the flywheels allow breaking for accurate landing. These robots are primarily designed for microgravity celestial bodies such as asteroids. The thruster-based designs use a single (Kim et al., 2016) or multiple thrusters (Tonasso et al., 2024) to perform the hop. Two interesting design concepts for tensegrity hopping robots were proposed in Kim et al. (2016) that can achieve thruster-orientation control using gimbals and cable actuation. The first design concept is to enclose the tank and thruster nozzle inside a two-degree-of-freedom gimbal structure, which allows adjustment of the thruster orientation for hopping. However, due to its environment, it cannot move its whole body, e.g., when the robot is stuck in a crater. The second concept is a cable-actuated thruster system which leverages the shape-shifting capability of the outer tensegrity structure and the high degree of freedom present in an active tensegrity robotic probe. In this system, the thruster and the payload are connected to the ends of the tensegrity rods by fixed-length compliant cables. By changing the structure’s shape, orientation control can be achieved within predefined angle limits.

Focusing on the hopping trajectory shaping, the initial launching errors, the uncontrolled ballistic phase, and undesired after-landing motions (e.g., bouncing, tumbling, slipping, etc.) take the hopping robot away from the desired landing site and might be in forbidden zones. Two possible solutions were proposed in the literature to overcome the after-landing motions. Considering the known surface properties, the first type of solution defines feasible take-off and landing cones and generates feasible hopping trajectories satisfying the defined cone constraints (Campana and Laumond, 2016; Upadhyay and Aguiar, 2020). By doing so, the slipping and bounded impact speed and terrain avoidance constraints can be addressed; however, the bouncing and tumbling motions cannot be controlled. Hence, this solution may be limited to terrestrial robots with instantaneous hopping capabilities (Haldane et al., 2017). Moreover, the take-off and landing cone constraints are coupled and may lead to non-existence of solutions. The second category of solutions uses internal flywheels to change the robot’s attitude in flight such that the robot falls over its face, leading to reduced bouncing. Then, breaking was applied to reduce the robot velocity (Li et al., 2020). The performance of this approach depends on the accuracy of the landing surface information and landing site selection for the hopping robot. On the other hand, the initial launching errors and the uncontrolled ballistic phase were rarely addressed and realized by the probability distribution of random errors (Upadhyay and Aguiar, 2020; Hockman and Pavone, 2020; Liang et al., 2022) to provide a worst-case landing region for validation of solutions as mentioned earlier.

In Tonasso et al. (2024), a lunar drone with thrusters combines ballistic trajectory with hovering. Here, the authors analysed the average thrust, total thrust duration, and required propellant for three types of trajectories that are (i) ballistic, (ii) a constant altitude flight, and (iii) mixed ballistic and constant altitude flight, which shows a controlled landing. However, the trajectories were pre-planned, and no active inflight trajectory shaping control was exercised. The in-flight trajectory generation or shaping using guidance theory is a widely researched topic in missile guidance problems (Ohlmeyer and Phillips, 2006; Mondal and Padhi, 2018; Ryoo et al., 2005), which has the capability to change the impact angle (Ratnoo and Ghose, 2008; Kumar and Ghose, 2017) and hence the potential to address the hopping motion issues. However, this approach is scarce in the hopping robot literature, which will be the key focus of this work.

This work demonstrates that impact angle-based trajectory shaping is a potential solution to the accurate landing problems (initiated launching errors, uncontrolled ballistic phase, and undesired after-landing motions) of hopping robots. The key contributions of this work are as follows:

• This work proposes a real-time trajectory shaping control using impact angle-based guidance that enables a thruster-based hopping robot to perform in-flight trajectory correction satisfying the landing objectives. Realising the guidance command to shape the trajectory using a tensegrity hopping robot.

• In contrast to the existing work, this is the first work on hopping robots (in the authors’ knowledge) that analyses the effect of impact angles (angles at the landing time on a surface) on the robot’s after-landing deviation due to sliding and bouncing motions.

• The proposed guidance algorithm is derived using optimal control theory and generates an optimal trajectory, minimising the control effort for each achievable impact angle.

• The effectiveness of the proposed approach with extensive simulation by using two cases of different impact angles and angled surfaces.

• The Generalized Vector Explicit (GENEX) guidance is used in the context of trajectory shaping of a hopping robot for the first time.

The remainder of the paper is as follows: the concerned problem is described in Section 2. The proposed method, including the basics of impact angle-based guidance, is presented in Section 3, followed by the conceptual realisation of the proposal for a thruster-based hopping robot in Section 4. Section 5 presents the simulation results, and the paper is concluded in Section 6.

## 2 Problem description

**FIGURE 1:**
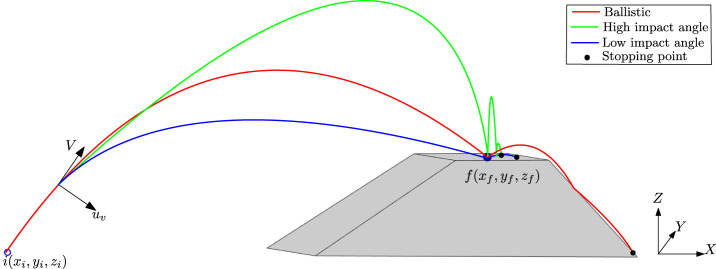
Hopping trajectory schematic.

Consider a point-mass robot hopping from a given initial position $i\left(x_{i},y_{i},z_{i}\right)$ to a given final position $f\left(x_{f},y_{y},z_{f}\right)$ on a terrestrial body surface, as shown in Figure 1. The surface is modelled using a polygonal mesh (Ettlin and Bleuler, 2006; Upadhyay and Aguiar, 2020), where 3D position coordinates of the surface data are used to create the triangulation mesh composition of tilted polygonal patches and horizontal patches (flat on the X-Y plane) as shown in Figure 1 by the grey-shaded region. As discussed in Ettlin and Bleuler (2006), each patch of the mesh has its specific material properties (e.g., coefficient of restitution), which are calculated by finding the mean of all respective 3D points material values under that polygonal patch. Hence, the coefficient of restitution μ for each patcisre considered to be known. Taking into account the negligible sideways disturbances and motion during flight, the robot heading angle ψ is kept constant so that the hopping motion takes place in the vertical X−Z plane in which the surface is characterised by a polyline of zero and finite slopes (Upadhyay and Aguiar, 2020). The hopping motion dynamics for the robot in the velocity frame can be defined as

$$\begin{array}{ll} \dot{V} & =-g\sin\left(\gamma \right)\\ \dot{\gamma } & =\frac{u_{v}-g\cos\left(\gamma \right)}{V}\\ \dot{\psi } & =0 \end{array}$$ (1)

where V , g , γ , and $u_{v}$ are the robot velocity, gravitational constant, flight path angle, and lateral acceleration along the vertical plane. Please note that $g=\text{eff}*g_{e}$ where eff is the fraction of earth’s gravity $g_{e}$ . In the 2-D hopping motion plane, the trajectory velocity is controlled by γ using $u_{v}$ . The initial and final values of γ are $\gamma_{i}$ and $\gamma_{f}$ , respectively. The robot velocity will change according to the ballistic model in Equation 1. The kinematics are written in the inertial coordinate frame as follows:

$$\begin{array}{ll} \dot{X} & =V\cos\left(\gamma \right)\cos\left(\psi \right)\\ \dot{Y} & =V\cos\left(\gamma \right)\sin\left(\psi \right)\\ \dot{Z} & =V\sin\left(\gamma \right) \end{array}$$

### 2.1 Bouncing motion characterization with surface interaction

With the given coefficient of restitution μ and the landing polygon slope m , the after-landing bouncing motion is computed from Equation 1 by considering $u_{v}=0$ and a new decreased initial velocity $V^{\prime }=\mu V$ while considering the bouncing angle the same as the impact angle for the flat surface with the sign change. The bouncing angle for the inclined polygon with a positive (negative) slope can be computed by adding (subtracting resp.) the landing polygon slope in the impact angle.

### 2.2 Requirements

1. The trajectory should be optimal. It can be mentioned that since the robot needs to fire thrusters during flight to control the trajectory, the guidance commands must be generated in an optimal control framework to minimise energy spending.
2. The hopping robot needs to start and finish at specific locations (usually called waypoints). It is important to note that there are mentions about the attitude control of the hopping robot to land in a way such that the after-landing motion is minimised. However, there are requirements for maintaining a specific attitude, such as pointing the camera at a specific angle or orientation for exploration or taking pictures. Therefore, changing attitudes can interrupt such operations. A trajectory-shaping impact angle control strategy (angle orientation of velocity vector during landing) can solve the issue. Hence, the impact angle is a requirement.
3. The trajectory shaping guidance algorithm must be generated in real-time, so it should have a closed-form expression.

We assumed that all the system states are measurable. Also, we have not considered any sensor or measurement noises.

## 3 Method: Generalized Vector Explicit (GENEX) guidance based impact angle control approach

The Genex algorithm has optimality, closed-form solution, and real-time execution (Ohlmeyer and Phillips, 2006) and hence is investigated as a potential candidate for the defined problem requirements 2.1. It can be mentioned that GENEX is used mainly as a missile guidance algorithm (Mondal and Padhi, 2018; Mondal and Padhi, 2019), path planning and collision avoidance problem (Subies Hueso et al., 2023) In this study, we will use GENEX to shape the trajectory of the hopping robot. We have introduced the philosophy and implementation facts in this section.

### 3.1 Basics of generalized vector explicit guidance (GENEX)

In this section, we present an overview of the guidance algorithm GENEX in the context of the interception of a target which is adopted from Ohlmeyer and Phillips, 2006. GENEX is formulated in an optimal control framework. It can guide an interceptor missile to a predefined position in space (generally called Predicted Interception Point or PIP) with its velocity vector oriented at a specific angle relative to the target’s velocity vector (called Impact angle). The guidance command has a closed-form expression, is computationally inexpensive, and easily implementable (Ohlmeyer and Phillips, 2006; Mondal and Padhi, 2019).

Let the lateral separation between an initial and its final point (PIP) be $y=y_{f}-y_{i}$ and define the zero effort miss (ZEM) as

$$z=y_{f}-y_{i}-{\dot{y}}_{i}T$$

where, $y_{f}$ and $y_{i}$ are constant, and $T=t_{f}-t$ is the time to go. Let v be the difference between the current velocity and the desired final velocity.

$$v={\dot{y}}_{f}-{\dot{y}}_{i}$$

Define the states as.

$$X_{1}=z=y_{f}-y_{i}-{\dot{y}}_{i}T$$

$$X_{2}=v={\dot{y}}_{f}-{\dot{y}}_{i}$$

Where.

$$\dot{z}=-uT$$

$$\dot{v}=-u$$

subject to the terminal conditions z=0 and v=0 at T=0 . The state equations may be written as

$$\left[\begin{array}{c} \dot{z}\\ \dot{v} \end{array}\right]=\left[\begin{array}{cc} 0 & 0\\ 0 & 0 \end{array}\right]\left[\begin{array}{c} z\\ v \end{array}\right]+\left[\begin{array}{c} -T\\ -1 \end{array}\right]u$$

or

$$\dot{X}=AX+bu,\;A=0,\;b=\left[\begin{array}{c} -T\\ -1 \end{array}\right]$$ (2)

where X is the state u is the control, and b may be time-varying. $X\left(t_{0}\right)=X_{0},X\left(t_{f}\right)=X_{f}$ . Select a cost function of the form

$$J=\overset{T_{0}}{\underset{0}{\int }}\frac{u^{2}}{2T^{n}}dT$$ (3)

n is an integer ≥0 . Equation 3 is a generalization of the standard integral of control energy cost in which the inclusion of T n in the denominator allows greater weight to be placed on the control usage as T→0 . The effect becomes stronger as n becomes more positive.

The Hamiltonian is defined by the scalar function

H=L+λf

where, $L=\frac{u^{2}}{2T^{n}}$ , f=AX+bu , and $\lambda =\left[\lambda_{1},\;\lambda_{2},\ldots ,\;\lambda_{n}\right]$ . The costate equations are

$$\dot{\lambda }=-\frac{\partial H}{\partial X}$$

the minimum principle of Pontryagin states that the control u is optimal when the Hamiltonian is minimized. Thus, to find the optimal control $u^{*}$ , set

$$\frac{\partial H}{\partial X}=0$$

Applying the minimum principle to the system, we get the following.

$$L=\frac{u^{2}}{2T^{n}}$$

$$H=L+\lambda f=\frac{u^{2}}{2T^{n}}+\lambda \left(AX+bu\right)$$

$$\frac{d\lambda }{dT}=-\dot{\lambda }=\frac{\partial H}{\partial X}=\lambda A$$ (4)

$$\frac{\partial H}{\partial u}=\frac{u}{T^{n}}+\lambda b$$

And

$$u^{*}=-\lambda bT^{n}$$ (5)

Now define a fundamental matrix M(T) that satisfies

$$\frac{dM}{dT}=MA,\;M\left(T=0\right)=I$$ (6)

Equations 4, 6 imply

λ=cM (7)

where c is a constant row vector. Then substitute (Equation 7) into (Equation 5) to obtain

$$u^{*}=-c\lambda bT^{n}$$

The state equation may be rewritten as

$$\frac{dX}{dT}=-AX-bu$$ (8)

Now consider the equation

$$\frac{d\left(MX\right)}{dT}=\frac{dM}{dT}X+M\frac{dX}{dT}$$ (9)

Substituting (Equations 6, 8) in Equation 9 gives

$$\frac{d\left(MX\right)}{dT}=MAX+M\left(-AX-bu\right)=-Mbu$$ (10)

c is a row vector and b is a column vector. Therefore, we have

$$cMb={\left(Mb\right)}^{T}c^{T}=\text{scalar}$$

Therefore, we can write

$$u^{*}=-{\left(Mb\right)}^{T}c^{T}T^{n}$$ (11)

Now substitute (Equation 11) into (Equation 10) and integrate:

$$\frac{d\left(MX\right)}{dT}=\left(Mb\right){\left(Mb\right)}^{T}c^{T}T^{n}$$

$$\overset{T}{\underset{0}{\int }}d\left(MX\right)=\left[\overset{T}{\underset{0}{\int }}\left(Mb\right){\left(Mb\right)}^{T}T^{n}dT\right]c^{T}$$

because c is a constant vector. Define the function

$$Q\left(T\right)=\overset{T}{\underset{0}{\int }}\left(Mb\right){\left(Mb\right)}^{T}T^{n}dT$$

Since A=0 (Please see Equation 2) we again have M=I , which gives

$$Q\left(T\right)=\overset{T}{\underset{0}{\int }}{bb}^{T}T^{n}dT$$

Using

$${bb}^{T}=\left[\begin{array}{cc} T^{2} & T\\ T & 1 \end{array}\right]$$

we obtain

$$Q=\overset{T}{\underset{0}{\int }}\left[\begin{array}{cc} T^{n+2} & T^{n+1}\\ T^{n+1} & T^{n} \end{array}\right]dT=\left[\begin{array}{cc} \frac{T^{n+3}}{n+3} & \frac{T^{n+2}}{n+2}\\ \frac{T^{n+2}}{n+2} & \frac{T^{n+1}}{n+1} \end{array}\right]$$

The inverse of Q may be obtained as

$$Q^{-1}=\frac{\left(n+1\right){\left(n+2\right)}^{2}\left(n+3\right)}{T^{2n+4}}\left[\begin{array}{cc} \frac{T^{n+1}}{n+1} & \frac{T^{n+2}}{n+2}\\ \frac{T^{n+2}}{n+2} & \frac{T^{n+3}}{n+3} \end{array}\right]$$

The optimum control is given by

$$u^{*}=-{\left(Mb\right)}^{T}Q^{-1}MXT^{n}=\left[T\;1\right]Q^{-1}T^{n}X$$

Let

$$\left[T\;1\right]Q^{-1}T^{n}=\left[C_{1}\;C_{2}\right]$$

Then

$$u^{*}=\left[C_{1}\;C_{2}\right]\left[\begin{array}{c} z\\ v \end{array}\right]$$

where.

$$C_{1}=\left(n+2\right)\left(n+3\right)/T^{2}$$

$$C_{2}=-\left(n+1\right)\left(n+2\right)/T$$

Now define new gains:

$$K_{1}=\left(n+2\right)\left(n+3\right)$$

$$K_{2}=-\left(n+1\right)\left(n+2\right)$$

Finally, the optimal control expression is given by

$$u_{Y}^{*}=\frac{1}{T^{2}}\left[K_{1}\left(y_{f}-y_{i}-{\dot{y}}_{i}T\right)+K_{2}\left({\dot{y}}_{f}-{\dot{y}}_{i}\right)T\right]$$

Similar control expressions are obtained in the inertial ‘ X ’ and ‘ Z ’ axes, i.e., $u_{X}^{*},u_{Z}^{*}$ . Putting them together, the control vector in the inertial coordinate frame becomes

$$U^{I}=\frac{1}{T^{2}}\left[K_{1}\left(R_{f}-R_{i}-V_{i}T\right)+K_{2}\left(V_{f}-V_{i}\right)T\right]$$

For simplicity, we will represent the control vector as

$$U^{I}={\left[U_{X}\;U_{Y}\;U_{Z}\right]}^{T}$$

where $U_{X}=u_{X}^{*},U_{Y}=u_{Y}^{*}$ , and $U_{Z}=u_{Z}^{*}$ are acceleration components generated along the inertial axis X,Y , and Z respectively.

**FIGURE 2:**
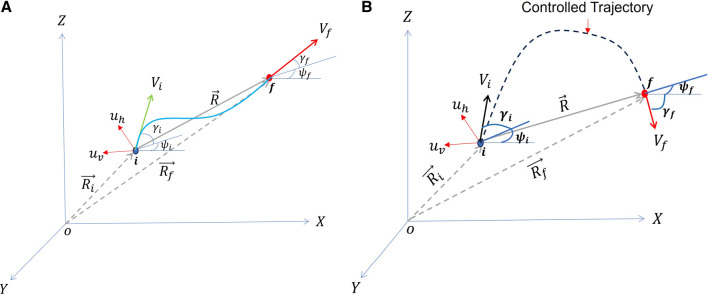
GENEX guides a vehicle from i to f . It also orients the velocity vector at the final point B with angles $\gamma_{f}$ and $\psi_{f}$ . **(A)** Missile-target engagement scenario. **(B)** Hopping robot guidance scenario.

The schematic of an engagement geometry is shown in Figure 2A. The GENEX guidance guides the interceptor from an initial position $i\left(X_{i}\;Y_{i}\;Z_{i}\right)$ to a fixed final position $f\left(X_{f}\;Y_{f}\;Z_{f}\right)$ . $R_{i}\left({\vec{R}}_{i}\right)$ , $R_{f}\left({\vec{R}}_{f}\right)$ denote the position vectors from the centre of the inertial coordinate frame, i.e., O , to the initial and final position, respectively. $R\left(\vec{R}\right)$ denotes relative vector from i to f , i.e., $R_{f}-R_{i}=R$ . The initial velocity vector $V_{i}\left({\vec{V}}_{i}\right)={\left[{\dot{X}}_{i}\;{\dot{Y}}_{i}\;{\dot{Z}}_{i}\right]}^{T}$ (green, arrow in Figure 2A) at i is oriented with a flight path angle of $\gamma_{i}$ and heading angle $\psi_{i}$ . The desired orientation of the velocity vector $V_{f}\left({\vec{V}}_{f}\right)={\left[{\dot{X}}_{f}\;{\dot{Y}}_{f}\;{\dot{Z}}_{f}\right]}^{T}$ at the final point f is shown as a red arrow. The desired value of γ is $\gamma_{f}$ , i.e., $\gamma \left(t=t_{f}\right)=\gamma_{f}$ . The desired value of ψ is $\psi_{f}$ , i.e., $\psi \left(t=t_{f}\right)=\psi_{f}$ .

The control thus obtained usually consists of acceleration components along the inertial frame. These are realised by transferring to other frames, such as body or velocity frames, using a rotation matrix. For example, to transfer from inertial to velocity frame (see Figure 2A), we may use the rotation matrix T(γ,ψ) , which is a rotation of elevation angle (flight-path angle) γ and azimuth angle ψ (heading angle). The acceleration in the velocity frame will be

$$U^{I}=T\left(\gamma ,\psi \right)u$$

Considering no thrust, i.e., no acceleration along the velocity direction, we can apply the acceleration ( $u_{v}$ and $u_{h}$ in Figure 2A) normal to the velocity vector and rotate it from the initial to the desired orientation as described in Figure 2A. The generated trajectory is shown in blue colour. Please note that, in the absence of thrust, we can not control the magnitude of the velocity vector at the final position. Therefore, we can update the magnitude $V_{f}$ with the current velocity magnitude V .

### 3.2 Hopping trajectory using GENEX

The hopping scenario is similar to the missile guidance scenario. However, in the case of hopping, we do not consider the drag (see Section 2), and the final angle $\gamma_{f}$ (landing angle) is of a different range of values (see Figure 2B). The GENEX can guide the hopping robot from i to f and generate the lateral acceleration to satisfy the final angle requirement. It is important to note that, in the case of the missile, the lateral acceleration is generated as the aerodynamic force by deflecting the control surfaces. However, in the case of the hopping robot, we need thrusters to generate lateral acceleration. However, the thruster-based hopping robots are rare in the literature. In the following section, we will present a conceptual analysis of how to implement GENEX for a thruster-based hopping robot.

## 4 A conceptual realization of GENEX based trajectory shaping control for a thruster- based hopping robot

The trajectory shaping of the hopping robot can be achieved by firing the thruster to produce lateral acceleration. Now, the question is how to produce the lateral acceleration for a hopping robot using the thruster. To answer this question, We will look into the thruster orientation control using gimbals and cable actuation of Spherical Tensegrity Robots (Kim et al., 2016). Here, the authors proposed thruster-based hopping, which is made feasible by the lightweight and compliant nature of the tensegrity structure. They also developed suitable controls for changing thruster orientation while initiating each hop (not during the flight). Three high-level approaches for adjusting the thruster direction during a hopping event were investigated, i.e., a) gimbaled nozzle thruster, b) gimbal-enclosed thruster system, and c) cable-actuated thruster system. They adjusted the thruster direction to initiate a hop in a specific direction. We can refer to the example of a cable-actuated thruster system, which is a possible method for controlling the orientation of the thruster. In this system, the thruster and the payload are connected to the ends of the tensegrity rods by fixed-length compliant cables. By changing the shape of the structure, orientation control can be achieved. Please refer to Fig. 8 in Section 4 of Kim et al. (2016) for a visual representation of this system. In Kim et al. (2016), the minimum and maximum angles (both elevation and azimuth) are achieved by cable actuation (inner, outer, and both); hence, the thruster can be oriented within these ranges.

### 4.1 Attitude and thruster orientation control

This study only considers the hopping motion confined to the vertical X−Z plane. Thus, the guidance command is the lateral acceleration in the vertical plane ( $u_{v}$ in Equation 1), which is applied normally to the velocity vector and changes its direction. The thruster of the tensegrity robot can be fired to produce lateral acceleration. However, the thruster must be oriented in the perpendicular direction of the velocity vector. The required angle of thruster orientation may be outside the angle range achieved by cable actuation (given in Kim et al. (2016)). It may happen in the initial part of the trajectory for a few specific initial hopping angles (the same as the initial flight-path angles). However, this possibility is low because the cable actuation can cover most of the flight-path angle range. Even if the initial thruster orientation angles are outside the maximum cable actuation range, the flight path angle comes within the cable actuation angle range soon after the hopping starts (due to the applied control). In this case (i.e., when the required thruster orientation angle is more than the maximum cable actuation angle), we can make some attitude corrections to adjust the extra angle orientation of the thruster. This concept is illustrated with the following example.

**FIGURE 3:**
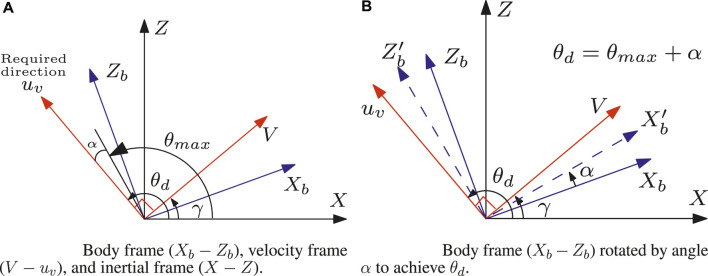
Different frames and their rotation to describe the thruster orientation of a point mass hopping robot when $\theta_{d}>\theta_{\max}$ . **(A)** Body frame (*X*_*b*_ − *Z*_*b*_), velocity frame (*V* − *u_v_*), and inertial frame (*X* − *Z*). **(B)** Body frame (*X_b_* − *Z*_b_) rotated by angle α to achieve *θ*_d_.

Here, we focus on Figure 3A. The body axis ( X−Z plane) is shown as $X_{b}$ and $Z_{b}$ (blue). The inertial frame is shown as X−Z (black). The velocity frame ( $V-u_{v}$ ; the inertial frame rotated by γ ; Its X direction is Velocity, and the Z axis is $u_{v}$ ) is shown in red. The flight path angle is γ , and the acceleration must be applied normally to the velocity vector. Therefore, the desired angle for the thruster direction is given by $\theta_{d}=\gamma +90^{0}$ . In Kim et al. (2016), we can see the maximum and minimum thruster elevation angles that can be achieved by actuating the cables. Let us denote these angles $\theta_{\max}$ (maximum) and $\theta_{\min}$ (minimum). If the desired thruster direction angle $\theta_{d}$ is less than $\theta_{\max}$ , i.e., $\theta_{d}<\theta_{\max}$ , the thruster can be oriented in the desired direction. However, we must consider the situation when $\theta_{d}>\theta_{\max}$ . In this case, the robot’s attitude correction is required. We have explained the required attitude control in Figures 3A, B. In Figure 3A, the required or desired thruster orientation of $u_{v}$ i.e., $\theta_{d}$ , and maximum angle orientation achievable, i.e., $\theta_{\max}$ are shown. Therefore, the body axis $\left(X_{b}-Z_{b}\right)$ should be rotated by angle $\alpha =\theta_{d}-\theta_{\max}$ to orient the thruster perpendicular to the velocity vector.

Let us consider the body axis $\left(X_{b}-Z_{b}\right)$ is rotated by angle $\alpha =\theta_{d}-\theta_{\max}$ to get a new body frame $\left(X_{b}^{\prime }-Z_{b}^{\prime }\right)$ as shown in Figure 3B (in blue dotted). The amount of rotation α helps achieve the desired thruster orientation (perpendicular to the velocity vector). We can have situations where the acceleration is opposite to what is shown in Figure 3A; that is, we can say that it is a negative acceleration $\left(-u_{v}\right)$ . In this case, we calculate the angles $\theta_{d}=-90{}^{\circ}+\gamma$ and $\alpha =-\left(\theta_{\min}+|\theta_{d}|\right)$ .

## 5 Results and discussions

We have assumed that attitude control was performed using any existing technique, that is, to rotate the body axis at an angle α as shown in Figure 3B. Therefore, the generated acceleration $u_{v}$ can be applied perpendicular to the velocity vector. Hence, we have focused on generating the hopping trajectory using the GENEX algorithm, not the attitude control. The performance of the proposed approach is evaluated in four scenarios with different initial and final conditions, as well as surface inclinations. For simulation, the gravitational acceleration is considered as $g=\text{eff}*g_{e}$ , where $g_{e}$ is the gravitational acceleration of Earth $9.8m/s^{2}$ . eff is the fraction of $g_{e}$ . In this study, we have considered eff=0.5 . The landing surface’s coefficient of restitution (μ) is 0.6. β is the inclination of the landing surface. The initial velocity of the robot is determined by its initial hopping angle $\gamma_{i}$ , starting coordinate $\left(x_{i},y_{i},z_{i}\right)$ , landing coordinate $\left(x_{f},y_{f},z_{f}\right)$ , and β . The distance between the current and the next waypoint is calculated by $d=\sqrt{\left({\left(x_{i}-x_{f}\right)}^{2}+{\left(y_{i}-y_{f}\right)}^{2}+{\left(z_{i}-z_{f}\right)}^{2}\right)}$ . The initial hopping velocity is calculated by $V=\sqrt{\left(dg\cos{\left(\beta \right)}^{2}/2\cos\left(\gamma_{i}\right)\sin\left(\gamma_{i}\right)\right)}$ . Therefore, the initial velocity varies with the initial hopping angle $\gamma_{i}$ . We considered $\left(x_{i},y_{i},z_{i}\right)=\left(30,20,0\right)m$ , $\left(x_{f},y_{f},z_{f}\right)=\left(50,50,0\right)m$ (for a flat surface), and $\left(x_{f},y_{f},z_{f}\right)=\left(50,50,3\right)m$ (for an inclined surface). We will study the robot’s bouncing when it lands on the surface with different slopes. Please note that we have assumed the robot to be a point-mass dynamics, and the angle (orientation) of the velocity vector at the time of landing (i.e., $\gamma_{f}$ ) is controlled. In the simulation, we stopped the bouncing when the robot’s velocity was less than 1m/s . The four cases are discussed as follows.

### 5.1 Case 1: Different $\gamma_{f}$ and the same $\gamma_{i}$

**FIGURE 4:**
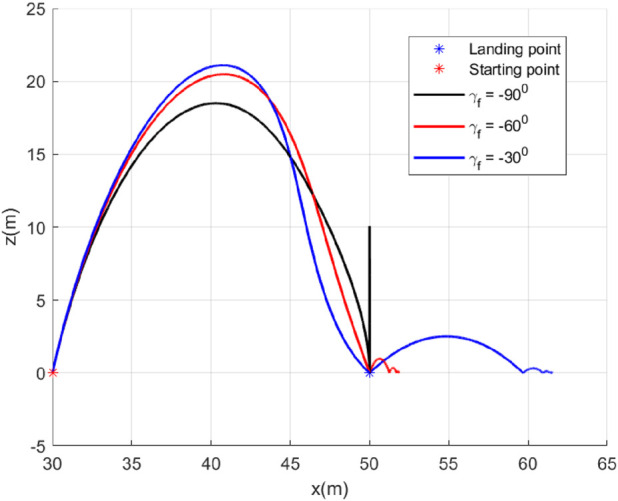
Trajectories (Case 1).

**FIGURE 5:**
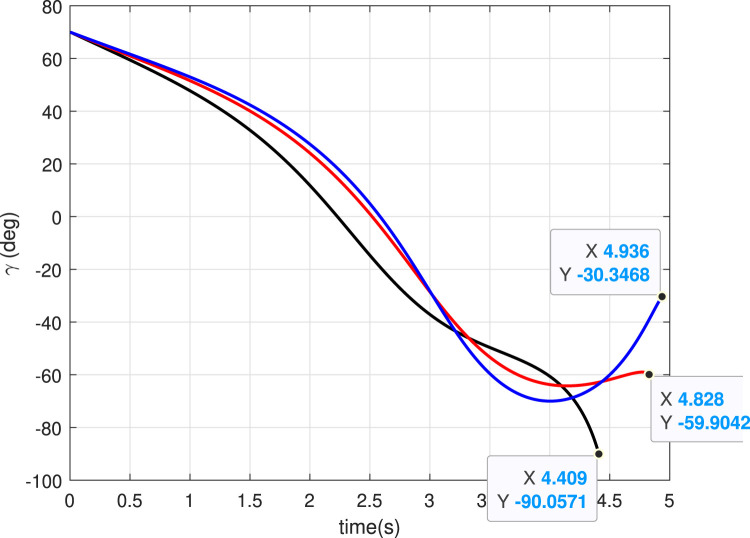
Angle histories (Case 1).

**FIGURE 6:**
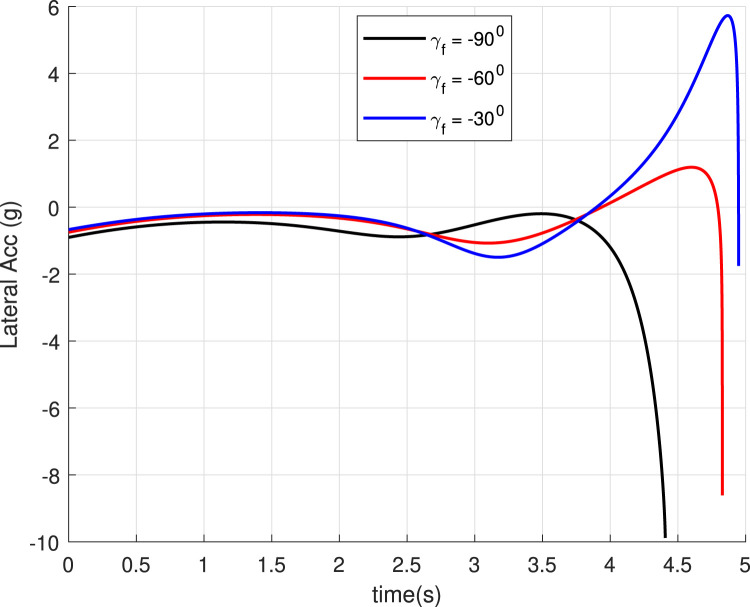
Lateral acceleration $\left(u_{v}\right)$ for different final angles (Case 1).

In this case, we consider a flat landing surface and the final landing angle $\gamma_{f}$ varies. We will study the robot’s bouncing when it lands on the surface with different orientations $\left(\gamma_{f}\right)$ of the velocity vector. This study is important because it can hint at selecting $\gamma_{f}$ while landing on a flat surface. We have selected three values for $\gamma_{f}$ . They are $\gamma_{f}=-90{}^{\circ},-60{}^{\circ},-30{}^{\circ}$ . The trajectories generated are shown in Figure 4. The minimum bounce is observed corresponding to the trajectory with $\gamma_{f}=-90{}^{\circ}$ . The robot settled almost at the landing point, which is expected. It happened due to the fact that the vertical component of the velocity was dominant (since $\gamma_{f}=-90{}^{\circ}$ ) when it landed, and there was practically no horizontal component. The horizontal component of the velocity starts increasing when the $\gamma_{f}$ is reduced to −60° and becomes dominant when $\gamma_{f}=-30{}^{\circ}$ . Figure 5 presents the flight-path angle history. The achieved angles at landing, i.e., final angle $\gamma_{f}$ , are marked. The values achieved justify the accuracy of the guidance GENEX. The lateral acceleration generated by the GENEX is shown in Figure 6, and the profile is smooth, which is desirable.

### 5.2 Case 2: Different $\gamma_{i}$ and same $\gamma_{f}$

**FIGURE 7:**
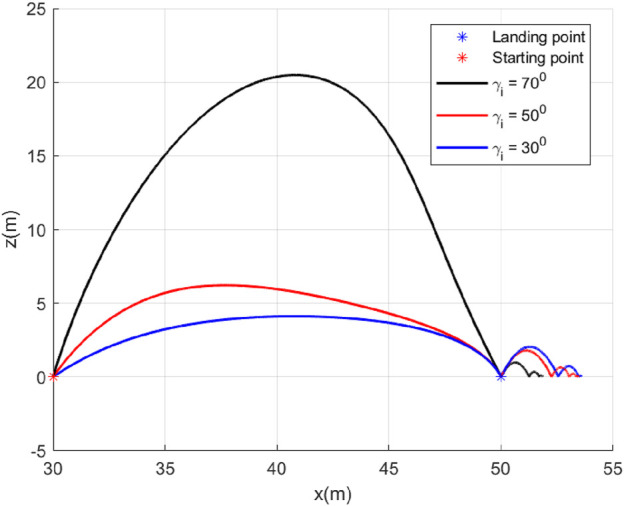
Trajectories (Case 2).

**FIGURE 8:**
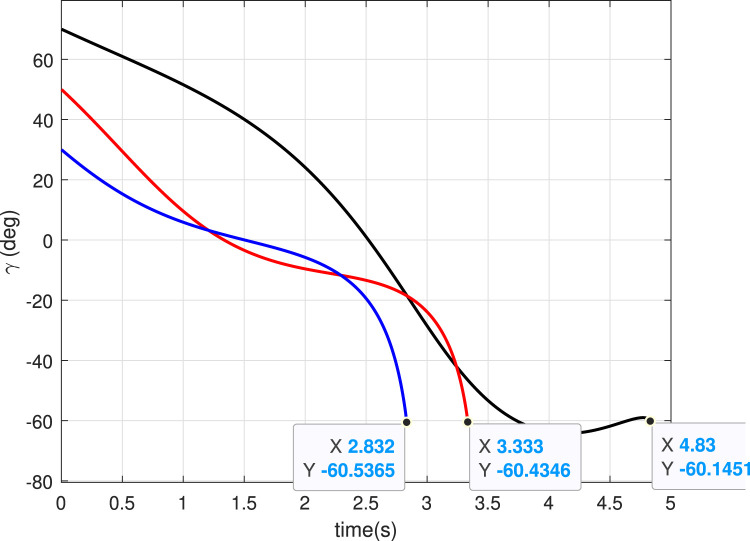
Angle histories (Case 2).

**FIGURE 9:**
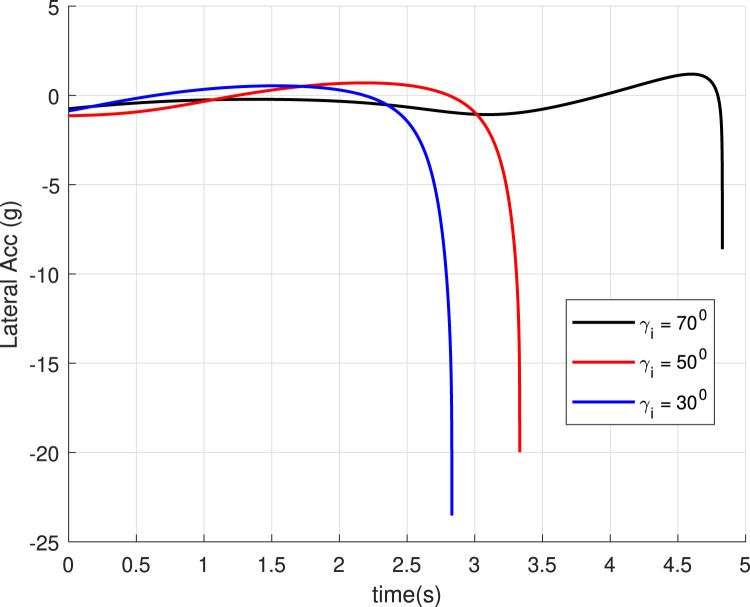
Lateral acceleration for different initial angles (Case 2).

In this case, we will study the effect of varying the initial hopping angle $\gamma_{i}$ , keeping the final angle $\gamma_{f}$ fixed.It also signifies that the guidance helps the robot land at the landing point with accurate $\gamma_{f}$ even if there is an error in the initial hopping angle. Trajectories are shown in Figure 7 with $\gamma_{i}=30{}^{\circ},50{}^{\circ},70{}^{\circ}$ and $\gamma_{f}=-60{}^{\circ}$ . The angle history (Figure 8) shows that the desired value of $\gamma_{f}$ is achieved. Also, the guidance algorithm generates a smooth acceleration profile (Figure 9), contributing to this achievement.

### 5.3 Case 3: Slope of landing surface varies

**FIGURE 10:**
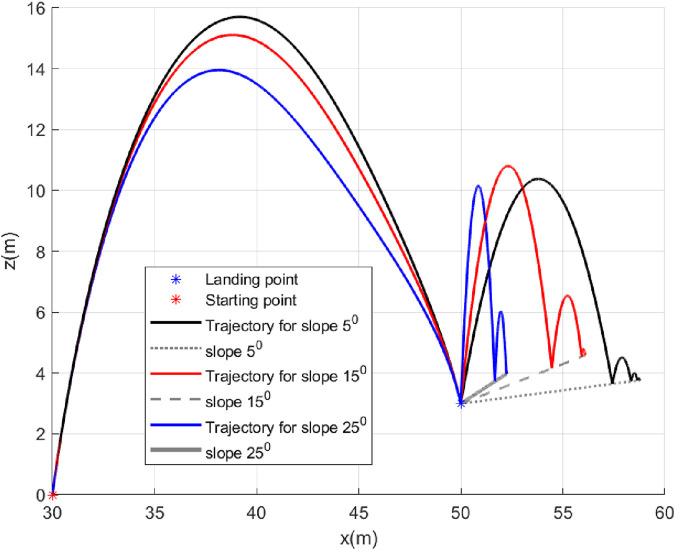
Trajectories (Case 3).

**FIGURE 11:**
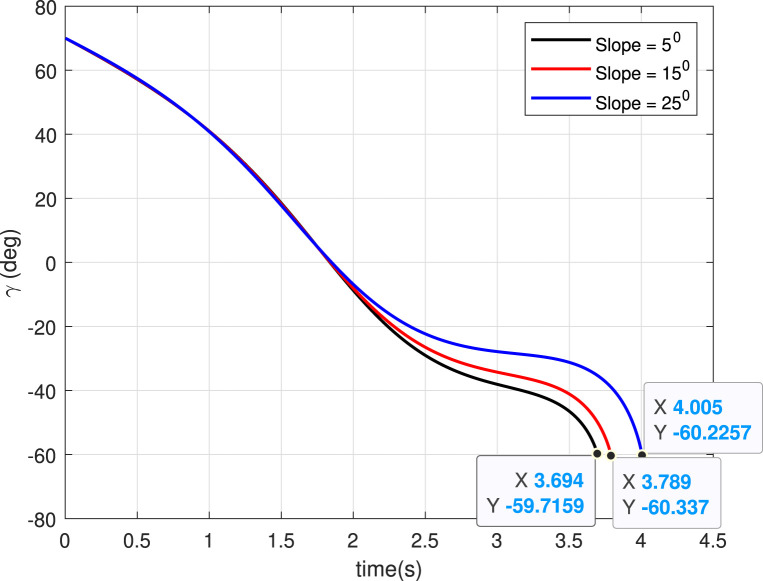
Angle histories (Case 3).

**FIGURE 12:**
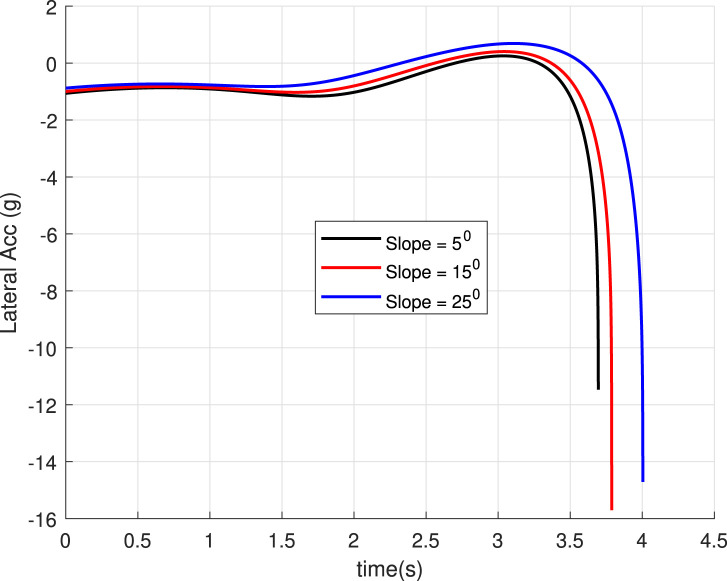
Lateral acceleration for the different inclination angles of the landing surface (Case 3).

In this case, we will study the bouncing of the robot on surfaces with different inclination angles (β) , keeping the $\gamma_{i}=70{}^{\circ}$ and $\gamma_{f}=-60{}^{\circ}$ fixed. This study is important in selecting the proper slope when multiple landing surfaces are available. The inclination angles were considered as β=5°,15°,25° and shown in grey with different line formatting (Figure 10). We can observe that bouncing from the landing point reduces with increasing slope of the landing surface, which is expected because the bounce back angle (measured from the horizontal plane) depends on the slope. The angle history plot (Figure 11) shows the $\gamma_{f}$ is achieved at the landing point. The acceleration profile generated (Figure 12) is smooth.

### 5.4 Case 4: Landing on same slope with different $\gamma_{f}$

**FIGURE 13:**
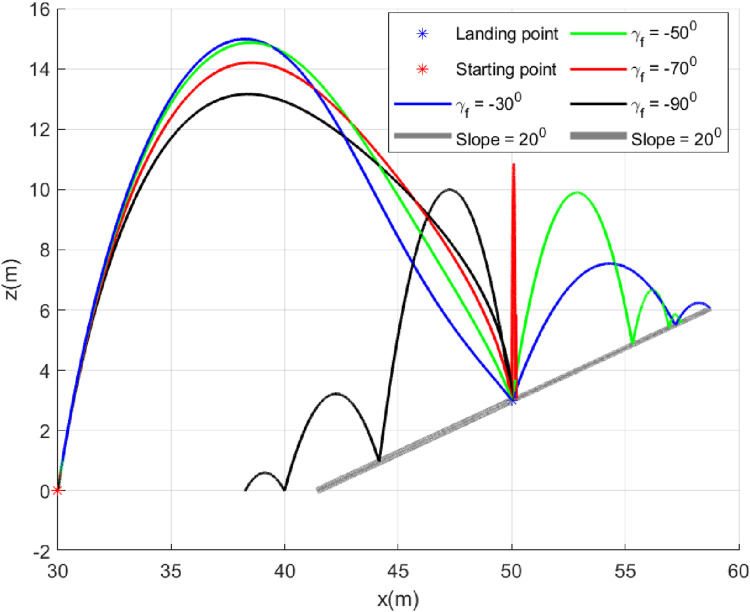
Trajectories (Case 4).

**FIGURE 14:**
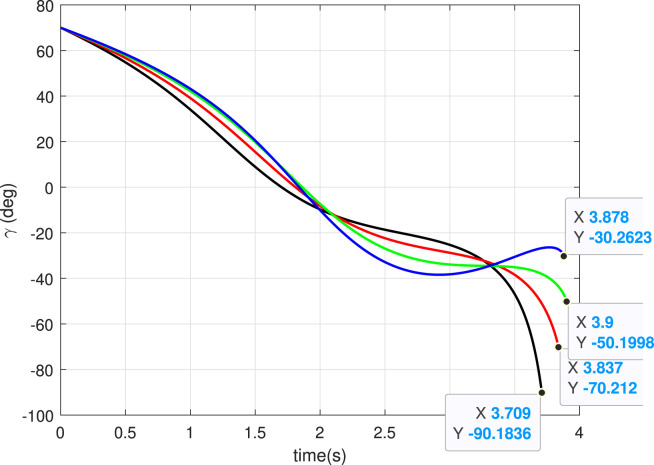
Angle histories (Case 4).

**FIGURE 15:**
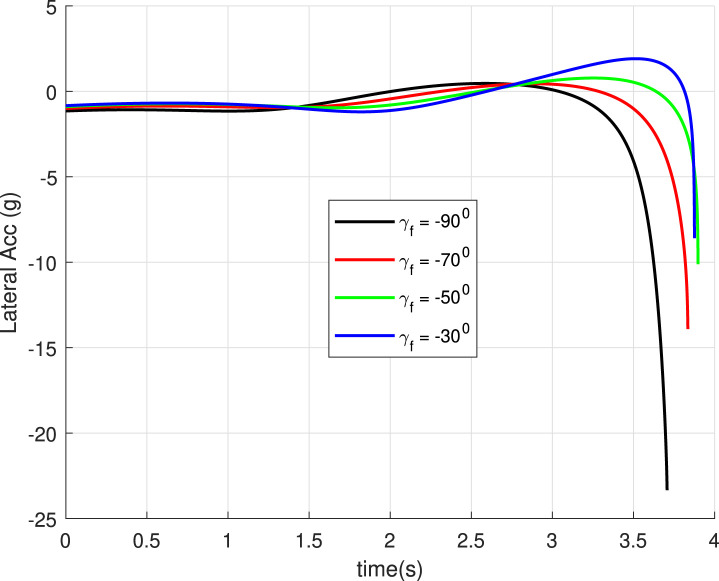
Lateral acceleration for different final angles and same inclination angle (Case 4).

We will study the bounce from a slope when the robot lands with different $\gamma_{f}$ . Most of the time, the landing waypoints along the path of the hopping robot remain fixed. Therefore, the slopes of the landing surfaces are also fixed. The significance of this study is to understand how the bouncing differs when the robot lands with different $\gamma_{f}$ on the same slope. It can also hint at selecting the $\gamma_{f}$ during the hop. However, we will provide the mathematical derivation of selecting optimal $\gamma_{f}$ in a separate manuscript. In Figure 13, the robot starts bouncing backwards down the slope when landed with $\gamma_{f}=-90{}^{\circ}$ (black trajectory). It continued bouncing when the slope ended, reaching a flat surface. It happened because the bounce-back angle is more than 90° (measured counterclockwise positive); the horizontal component is opposite to the hopping. On the other hand, the bounce in the forward direction (up along the slope) when the $\gamma_{f}>-70{}^{\circ}$ . Two cases were shown where $\gamma_{f}=-30{}^{\circ},-50{}^{\circ}$ . At $\gamma_{f}=-70{}^{\circ}$ , the bounce-back angle from the surface (measured counterclockwise positive) reaches almost near 90° , and the robot keeps bouncing almost at the landing point (red trajectory). Figure 14 shows the angle histories corresponding to the trajectories generated. It can be seen that the final goals are achieved successfully. The lateral acceleration generated is shown in Figure 15, and they are smooth.

## 6 Conclusion

We have proposed an optimal trajectory shaping control approach using GENEX for a thruster-based hopping robot. The algorithm can make the hopping robot achieve different impact angles for the same initial conditions, improving the landing accuracy. The trajectory shaping control can make the hopping robot land accurately at the landing position with a precalculated impact angle despite any initial hopping angle or velocity errors. Thus, the analysis of landing inaccuracy is relaxed. We have presented an example of the conceptual realization of the proposed approach that can be implemented on existing thruster-based hopping robot designs. The effectiveness of the proposed guidance is explained through a simulation study using various cases like horizontal and inclined landing surfaces with various initial final conditions. The results demonstrate the effect of landing angles on after-landing inaccuracy, which can be reduced by choosing the appropriate landing angle. The selection of the best landing angle for pinpointing, implementing realistic hopping robot models, and integration with a sequential multi-hopping trajectory generation approach are immediate works in the future. Another interesting future works are a study on the effect of attitude knowledge inaccuracies (such as biases and drift from onboard sensors) on the proposed approach and the integration of the proposed approach with an appropriate estimator to handle such inaccuracies are future works.

## Data Availability

The raw data supporting the conclusions of this article will be made available by the authors, without undue reservation.

## References

[B1] Burdick J. Fiorini P. (2003). Minimalist jumping robots for celestial exploration. Int. J. Robotics Res. 22, 653–674. 10.1177/02783649030227013

[B2] Campana M. Laumond J. P. (2016). Ballistic motion planning. 2016-Novem, 1410–1416. 10.1109/IROS.2016.7759230

[B3] Ettlin A. Bleuler H. (2006). “Rough-terrain robot motion planning based on obstacleness,” in 2006 9th International Conference on Control, Automation, Robotics and Vision, China, 5-8 Dec. 2006, 1–6. 10.1109/icarcv.2006.345116

[B4] Geromichalos D. Azkarate M. Tsardoulias E. Gerdes L. Petrou L. Perez Del Pulgar C. (2020). Slam for autonomous planetary rovers with global localization. J. Field Robotics 37, 830–847. 10.1002/rob.21943

[B5] Haldane D. W. Yim J. K. Fearing R. S. (2017). “Repetitive extreme-acceleration (14-g) spatial jumping with salto-1p,” in IEEE/RSJ International Conference on Intelligent Robots and Systems (IROS), USA, 1-5 Oct. 2023, 3345–3351. 10.1109/IROS.2017.8206172

[B6] Hockman B. Pavone M. (2020). “Stochastic motion planning for hopping rovers on small solar system bodies,” in Robotics research: the 18th international symposium ISRR (Germany: Springer), 877–893.

[B7] Hockman B. J. Frick A. Reid R. G. Nesnas I. A. Pavone M. (2017). Design, control, and experimentation of internally-actuated rovers for the exploration of low-gravity planetary bodies. J. Field Robotics 34, 5–24. 10.1002/rob.21656

[B8] Kim K. Chen L.-H. Cera B. Daly M. Zhu E. Despois J. (2016). “Hopping and rolling locomotion with spherical tensegrity robots,” in 2016 IEEE/RSJ international conference on intelligent robots and systems (IROS), China, 9-14 Oct. 2016 (IEEE), 4369–4376.

[B9] Kumar S. R. Ghose D. (2017). Three-dimensional impact angle guidance with coupled engagement dynamics. Proc. Institution Mech. Eng. Part G J. Aerosp. Eng. 231, 621–641. 10.1177/0954410016641442

[B10] Li X. Sanyal A. K. Warier R. R. Qiao D. (2020). Landing of hopping rovers on irregularly-shaped small bodies using attitude control. Adv. Space Res. 65, 2674–2691. 10.1016/j.asr.2020.02.029

[B11] Liang Z. Lv C. Zhu S. Ge D. (2022). Guidance for precision landing on asteroid using active hopping trajectory. Acta Astronaut. 198, 320–328. 10.1016/j.actaastro.2022.06.003

[B12] Mondal S. Padhi R. (2018). Angle-constrained terminal guidance using quasi-spectral model predictive static programming. J. Guid. Control, Dyn. 41, 783–791. 10.2514/1.g002893

[B13] Mondal S. Padhi R. (2019). Generalized explicit guidance with optimal time-to-go and realistic final velocity. Proc. Institution Mech. Eng. Part G J. Aerosp. Eng. 233, 4926–4942. 10.1177/0954410019834780

[B14] Ohlmeyer E. J. Phillips C. A. (2006). Generalized vector explicit guidance. J. Guid. Control, Dyn. 29, 261–268. 10.2514/1.14956

[B15] Ratnoo A. Ghose D. (2008). Impact angle constrained interception of stationary targets. J. Guid. Control, Dyn. 31, 1817–1822. 10.2514/1.37864

[B16] Ryoo C.-K. Cho H. Tahk M.-J. (2005). Optimal guidance laws with terminal impact angle constraint. J. Guid. Control, Dyn. 28, 724–732. 10.2514/1.8392

[B17] Subies Hueso J. Mondal S. Tsourdos A. Chadwick A. (2023). Real-time collision avoidance trajectory planner using generalized vector explicit guidance. AIAA 2023-1734. 10.2514/6.2023-1734

[B18] Tonasso R. Tataru D. Rauch H. Pozsgay V. Pfeiffer T. Uythoven E. (2024). A lunar reconnaissance drone for cooperative exploration and high-resolution mapping of extreme locations. Acta Astronaut. 218, 1–17. 10.1016/j.actaastro.2024.02.006

[B19] Tzanetos T. Bapst J. Kubiak G. Tosi L. P. Sirlin S. Brockers R. (2022). “Future of mars rotorcraft - mars science helicopter,” in 2022 IEEE Aerospace Conference (AERO), China, 5-12 March 2022, 1–16. 10.1109/AERO53065.2022.9843501

[B20] Upadhyay S. Aguiar A. P. (2020). Constrained hopping traversability analysis on non-uniform polygonal chains. IEEE Access 8, 36691–36701. 10.1109/ACCESS.2020.2975114

